# Elective Surgery for Diverticulitis in Swiss Hospitals

**DOI:** 10.3389/fsurg.2021.717228

**Published:** 2021-10-12

**Authors:** Seraina Faes, Martin Hübner, Nicolas Demartines, Dieter Hahnloser, David Martin, The Swiss Snapshot Diverticulitis Group, Paolo Abitabile

**Affiliations:** Department of Visceral Surgery, Lausanne University Hospital CHUV, University of Lausanne (UNIL), Lausanne, Switzerland

**Keywords:** diverticulitis, management, Switzerland, elective surgery, laparoscopy

## Abstract

**Objective:** To assess current management of diverticulitis in Switzerland.

**Methods:** Prospective observational study of diverticulitis management and outcomes in surgical departments over a 3-month time period. Hospital category was graded according to the Swiss Medical Association (FMH) as: U: University; A: Cantonal; B: Regional; P: Private.

**Results:** 75 participating hospitals treated 1,015 patients, among whom 214 patients (21%) had elective sigmoid resections in 49 hospitals. Indication for elective resection were recurrent diverticulitis, previous complicated diverticulitis, fistulas, and stenosis. Surgeries were performed completely laparoscopically in 185 cases (86%) and required conversion to open in 19 cases (9%). Overall postoperative complication rate was 18% (*n* = 39) and no mortality was observed. Operation time, surgeons experience and hospital stay differed considerably between hospital categories.

**Conclusions:** Elective sigmoid resection for diverticulitis in Switzerland was mainly performed laparoscopically with low postoperative morbidity. Different practices and outcomes between institutions were observed.

## Introduction

Traditionally, surgical resection was offered after the 2nd or 3rd episode of simple acute diverticulitis, in order to prevent recurrence and more serious forms (1, 2). Cohort studies have shown that complications occurred during the first episode, and that preventive surgery was not indicated (3, 4). Thus, diverticulitis management changed considerably during the last decade with a more conservative approach in the acute and in recurrent setting, due to evidence on the natural benign course of the disease (5). Minimal-invasive surgery without ostomy has definitely reduced morbidity, improved post-interventional quality of life and reduced costs, and seems now to be the treatment of choice (6). Two international guidelines on the management of diverticulitis were updated in 2020 (7, 8), however, there is often a gap of a few years for them to be applied universally.

The Swiss healthcare system is a public-private mix. Health care providers such as doctors and hospitals are partly private and public. In addition, care is the responsibility of the cantons, but certain aspects are regulated at the federal level. Every citizen is obligatorily covered by a basic insurance and pays monthly premiums to the health insurance of his choice. Basic insurance covers efficient and economical services which are defined by legal regulations. In order to supplement the basic insurance benefits, a citizen can take out an additional private insurance, which covers additional services in hospitals or private clinics, with free choice of location and caregiver. In principle, surgical residencies take place in University hospitals, and surgeons who work in public hospitals do not work in private hospitals.

Considering these variations in the health system and the latest published management recommendations, the aim of the present study was to assess practice and outcomes of elective surgery for diverticulitis among different institutions in Switzerland over a specific previous period.

## Materials and Methods

This study assessed secondary outcomes of a prospective observational Swiss Snapshot Diverticulitis study which assessed the in-hospital management of colonic diverticulitis in surgical departments in Switzerland over a 3-month time period in 2014 (9). Participating centers were asked to provide demographics, surgical details, and outcomes. The diagnosis had to be clarified, but the indication to operate was at the discretion of the surgeon. Simple diverticulitis was defined as peridiverticular inflammation of a colonic segment, and complicated forms included abscesses, perforations, fistulas, and occlusions. The type of procedure (resection +/− anastomosis +/− stoma) and the surgical approach (laparoscopy or open) had to be specified. Complications were graded according to the Clavien classification within 30 postoperative days (10). Major complications were defined as grade ≥3b. Hospital category was graded according to the Swiss Medical Association (FMH) as: U: University; A: Cantonal; B: Regional; P: Private.

Descriptive statistics for categorical variables were reported as number and percentage, while continuous variables were reported mean and standard deviation. Continuous variables were compared with the Kruskal–Wallis test, while categorial variables were compared with the Pearson's chi square test. A *p* ≤ 0.05 was considered statistically significant. Analyses performed using SPSS 26.0 software (SPSS Inc., Chicago, IL).

## Results

During study period, 75 participating hospitals treated 1,015 patients, among whom 214 patients (21%) had elective sigmoid resections in 49 hospitals. Indication for elective resection were recurrent diverticulitis, previous complicated diverticulitis, fistulas, and stenosis, which varied between categories of hospitals (Table 1). Most performed surgery was colon resection with anastomosis without protective ileostomy (*n* = 209, 98%). Surgeries were performed completely laparoscopically in 185 cases (86%) and required conversion to open in 19 cases (9%). The reasons for conversion were adhesions (*n* = 10), local inflammation (*n* = 5), inflammatory fistulas (*n* = 2), patient obesity (*n* = 1), and a ruptured suture line (*n* = 1). Nine complications occurred intraoperatively (4%), in particular spleen decapsulation (*n* = 3), anastomotic leakage at air test (*n* = 3), rectal perforation (*n* = 2), and small bowel perforation (*n* = 1).

**Table 1:** Patient demographics and surgical details according to hospital category.

	**University (*n* = 14)**	**Cantonal (*n* = 73)**	**Regional (*n* = 91)**	**Private (*n* = 36)**	***P*-value**
Age (years) (mean, SD)	64 (14)	60 (13)	61 (11)	65 (12)	0.425
BMI (kg/m^2^) (mean, SD)	27 (4)	27 (5)	27 (5)	25 (4)	0.191
Gender (M: F)	5:9	32:40	36:55	14:22	0.850
ASA score (I-II: III-IV)	11:3	63:10	79:12	29:7	0.718
Indication					**≤0.01**
Recurrent diverticulitis	4 (29%)	51 (70%)	61 (67%)	32 (89%)	
Perforated diverticulitis	3 (21%)	12 (16%)	17 (19%)	2 (5%)	
Stenosis	1 (7%)	0 (-)	6 (7%)	1 (3%)	
Fistula	5 (36%)	8 (11%)	5 (5%)	1 (3%)	
Other	1 (7%)	2 (3%)	2 (2%)	0 (-)	
Colon resection					0.241
Anastomosis without ostomy	14 (100%)	71 (97%)	88 (97%)	36 (100%)	
Anastomosis with ostomy	0 (-)	0 (-)	3 (3%)	0 (-)	
Hartmann procedure	0 (-)	2 (3%)	0 (-)	0 (-)	
Minimally invasive approach	10 (71%)	63 (86%)	76 (84%)	36 (100%)	0.175
Teaching	7 (50%)	14 (19%)	14 (15%)	0 (-)	**≤0.01**

*Significant p values (<0.05) are displayed in bold characters*.

*Continuous variables were compared with the Kruskal–Wallis test, while categorial variables were compared with the Pearson's chi square test*.

*SD, standard deviation; BMI, body mass index; ASA, American Society of Anesthesiologists*.

Overall postoperative complication rate was 18% (*n* = 39). Major complications occurred in eight patients (4%) and no mortality was observed. Surgical site infections occurred in 13 patients (6%), anastomotic leak in five patients (2%), bleeding in eight patients (4%), and postoperative ileus in five patients (2%).

**Figure 1:**
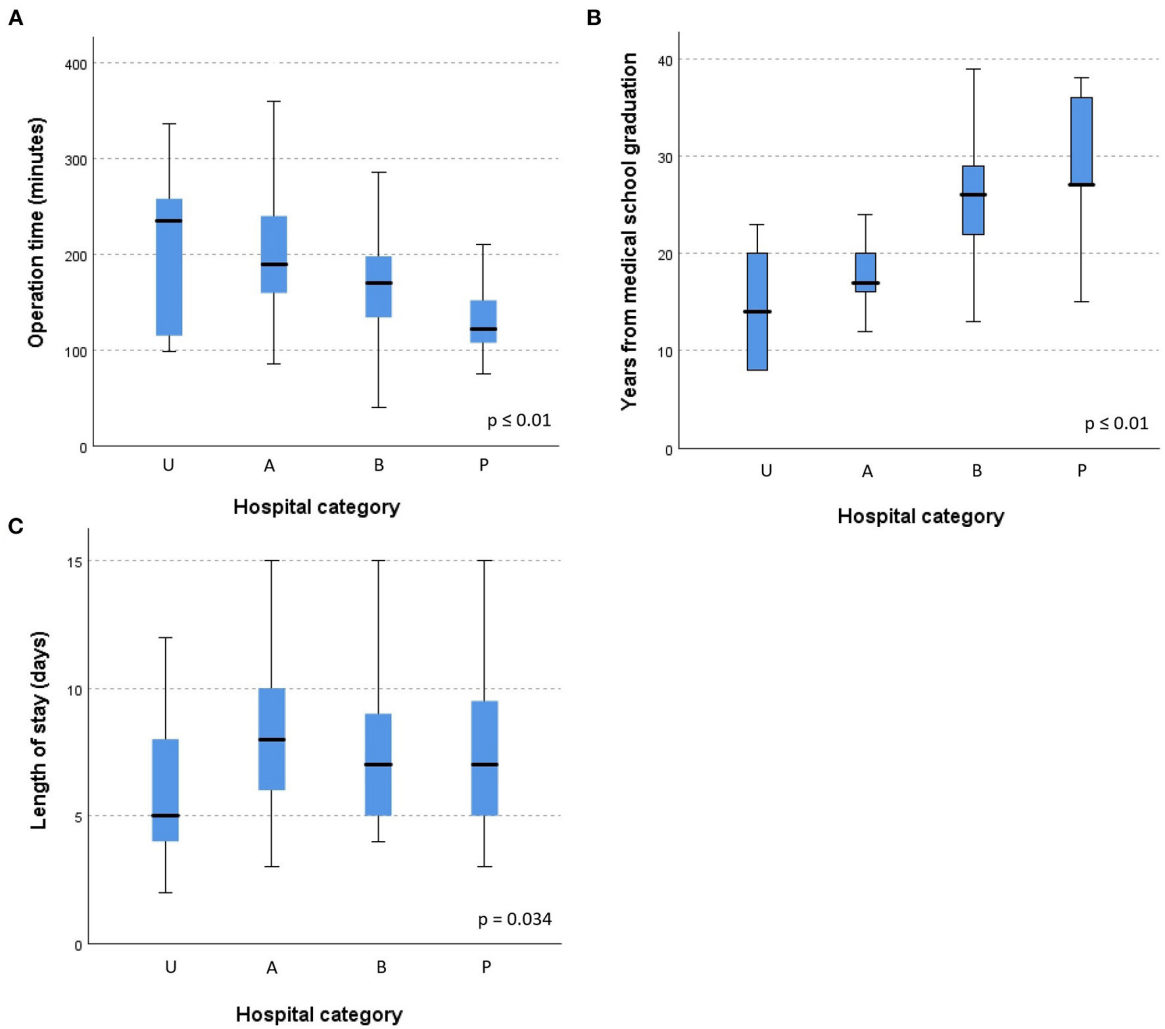
**(A–C)** Elective surgery for diverticulitis: operation time, surgeon's experience and hospital stay according to hospital category. Hospital category defined as: U, University; A, Cantonal; B, Regional; P, Private.

Operation time, surgeons experience and hospital stay differed considerably between hospital categories (Figure 1).

## Discussion

In this study, laparoscopic colonic resection without protective ileostomy appeared to be highly standardized (98%), regardless of the type of hospital. This is a validated approach with increasing popularity in recent years, and it seems indicated on patients with recurrent disease or persistent symptoms after uncomplicated and complicated diverticulitis in order to improve their quality of life (8, 11).

The aim of elective surgical treatment of diverticular disease is the removal of the disease with prevention of its recurrence and restoration of bowel continuity whenever possible (12). There are many studies that have assessed elective surgery, but they are very heterogeneous and of low quality, with a high probability of selection bias (13). The surgical indication must now be individualized for each patient, and based on the frequency of recurrence, the occurrence of complications (perforations, fistulas, strictures), the duration and severity of symptoms, as well as the patient's comorbidities (7, 8). The indication to operate on a patient after an episode of complicated diverticulitis should follow the same principles as for patients with uncomplicated diverticulitis, and resection is not routinely recommended. This personalized treatment could explain the differences in the number of patients, operative indications, operative times as well as length of stay that were observed among hospitals in the present study. Academic hospitals seemed to have a larger case mix with more complicated diverticulitis and fistulas, and operating surgeons were less experienced, which resulted in longer operative times. In addition, half of the interventions were taught. On the other hand, surgeons in private hospitals were more experienced and operated faster. It has been previously described that the duration of surgery, length of stay, and rates of intraoperative and postoperative complications decreased with increasing surgeon's experience (14). In fact, length of stay was shorter in academic hospitals in the present study, which may be explained by the standardized pathways (ERAS: Enhanced Recovery After Surgery) allowing to optimize perioperative care and outcomes.

Regarding the surgical approach, a systematic review of randomized controlled trials showed that there was insufficient evidence to support the efficacy and safety of laparoscopy compared to open surgery (15). However, in the event of elective resection, the laparoscopic approach should be preferred over open surgery, when feasible, since its short- and long-term benefits are probably the same as those demonstrated for other diagnostics (8). This way of proceeding was clearly demonstrated in this present study, where the vast majority of interventions (86%) were carried out by laparoscopy.

Several limitations of the present study need to be addressed. First, there may be a selection bias, which limits generalization. Indeed, more health-conscious surgeons may choose to participate in studies assessing practices and outcomes. The inclusion of qualitative aspects focusing on patient and care provider perspectives on the elective management of diverticulitis would have been interesting and should be planned in future research. Lastly, the study provides a glimpse of what was happening in 2014, but probably the practices are the same nowadays. Two international guidelines were updated in 2020 (7, 8), however, there is often a gap of a few years for them to be applied universally. Future studies should also look at what was done before and what was done after, and also integrate long-term follow-up.

In conclusion, elective sigmoid resection for diverticulitis in Switzerland was mainly performed laparoscopically with low conversion and complication rates, and different practices and outcomes between institutions were observed.

## Data Availability Statement

The raw data supporting the conclusions of this article will be made available by the authors, without undue reservation.

## Ethics Statement

The studies involving human participants were reviewed and approved by Commission cantonale d'éthique de la recherche sur l'être humain CER-VD, protocol 68/14. The patients/participants provided their written informed consent to participate in this study.

## Author Contributions

SF, MH, ND, DH, and DM: study design, manuscript writing, and critical revision of the manuscript. SF, MH, and DH: data collection. DM: statistical analysis. ND: supervision. All authors contributed to the article and approved the submitted version.

## Swiss Snapshot Diverticulitis Group

Swiss Snapshot Diverticulitis Group: Paolo Abitabile, Dritan Abrazhda, Michele Arigoni, Vahid Bakhshi-Tahami, Jean-Pierre Barras, Thomas Beck, Vincent Bettschart, Paul Biegger, Karin Bläuer, Stefan Breitenstein, Franziska Brinkmann, Lukas Brügger, Hans Brunner, Walter Brunner, Claude Bussard, Jean-Marie Calmes, Jean-Pierre Chevalley, Michael Chilcott, Denis Christinaz, Dimitri Christoforidis, Carlo Coduri, Nadine Crivelli, Aris D'Ambrogio, Branimir Damjanovic, Wiebke Decking, Diego De Lorenzi, Charles de Montmollin, Sona Deretti, Alexandre Descloux, Urs Diener, Marco Di Lazzaro, Luca Di Mare, Rok Dolanc, Andrea Donadini, Georg Donner, Bernhard Egger, Michel Erne, Fabrizio Fasolini, Charlotte-Ulrike Finkenzeller, Ivo Ralf Fischer, Daniel Frey, Raffaele Galli, Walter Gantert, Alain Garcia, Jörg Genstorfer, Pascal Gervaz, Bijan Ghavami, Nicola Ghisletta, Duri Gianom-Campell, Mauro Giuliani, Christine Glaser, Emanuel Gmür, Federico Goti, Jürg Gresser, Felix Grieder, Gerald Gubler, Adriano Guerra, Silvio Gujer, Jürg Gurzeler, Susanne Habelt, Peter Häfliger, Andres Heigl, Dominik Heim, Juliette Henri, Mark Henschel, Rudolf Herzig, Franc Hetzer, Henry Hoffmann, Markus Huber, Regula Humm, Adrienne Imhof, Daniel Inderbitzin, Manuel Jakob, Renata Jori, Philomena Kastner, Andreas Keerl, Ulf Kessler, Philipp Kirchhoff, Jennifer Klasen, Katrin Kleinschmidt, Jürg Knaus, Markus Koch, Michael Kodsi, Erwin Kohlberger, Stefan Kull, Beat Künzli, Sebastian Lamm, Stéphanie Laperrousaz, André Leuenberger, Patrick Mäder, Styliani Mantziari, Florian Martens, Lukas Marti, Olivier Martinet, Jean Mégevand, Gian Melcher, Antoine Meyer, Pierre Meyer, Philippe Morel, Murielle Mormont, Beat Muggli, Markus Müller, Stephan Müller, Andrew Munday, Surennaidoo Naiken, Antonio Nocito, Peter Nussbaumer, Daniel Oertli, Alexandre Paroz, Angelo Pelloni, Jörg Peltzer, Matthias Peter, Sebastian Pohle, Philippe Posso, Hervé Probst, Alexander Radke, Martin Reber, Luca Regusci, Verena Reichl, Andreas Remiger, Jean-Claude Renggli, Monika Richter, Paavo Rillmann, Frédéric Ris, Nadja Ristagno, Luca Rondi, Robert Rosenberg, Raffaele Rosso, Alend Saadi, Bernd Schenkluhn, Martin Schilling, Rolf Schlumpf, Bruno Schmied, Michael Schmitz, Rémi Schneider, Othmar Schöb, Claudio Soravia, René Spalinger, Rudolf Steffen, Daniel Steinemann, Reto Stocker, Ulrich Stricker, Alexander Stupnicki, Michel Suter, Daniel Tassile, Adrien Tempia, Derya Topal, Rebekka Troller, Daniel Trötschler, Cédric Vallet, Denise Vettorel, Carsten Viehl, Peter Villiger, Peter Vogelbach, Marco von Strauss und Torney, Stephan Vorburger, Matthias Walting, Markus Weber, Heinz Wehrli, Bernhard Widmann, Stefan Wildi, Alessandro Wildisen, Bernd Wilhelm, Mariano Winckler, Marc Worreth, Jörg Wydler, Sidika Yakarisik, Urs Zingg, Christof Zöllner, Markus Zuber, Michael Zünd.

## Conflict of Interest

The authors declare that the research was conducted in the absence of any commercial or financial relationships that could be construed as a potential conflict of interest.

## Publisher's Note

All claims expressed in this article are solely those of the authors and do not necessarily represent those of their affiliated organizations, or those of the publisher, the editors and the reviewers. Any product that may be evaluated in this article, or claim that may be made by its manufacturer, is not guaranteed or endorsed by the publisher.
